# Clinical Predictors of Neurological Outcome within 72 h after Traumatic Cervical Spinal Cord Injury

**DOI:** 10.1038/srep38909

**Published:** 2016-12-12

**Authors:** Zhi Qiu, Fangyong Wang, Yi Hong, Junwei Zhang, Hehu Tang, Xiang Li, Shudong Jiang, Zhen Lv, Shujia Liu, Shizheng Chen, Jiesheng Liu

**Affiliations:** 1 School of Rehabilitation Medicine, Capital Medical University, Beijing 100068, China; 2 Department of Spine Surgery, Beijing Bo’ai Hospital, China Rehabilitation Research Center, No. 10, North Jiaomen Road, Fengtai District, Beijing 100068, China

## Abstract

To investigate the prognostic values of clinical factors 72 h within traumatic cervical spinal cord injury (TCSCI). Data were extracted from the medical materials of 57 TCSCI cases. AIS was used as the outcome measure and divided into dichotomous variables by two methods, i.e. “complete(AIS = A)/incomplete(AIS ≠ A) SCI” and “motor complete(AIS = A or B)/incomplete(AIS ≠ A and B) SCI”. Relationships between evaluated factors and outcomes were investigated by univariate and multivariate methods. MRI Cord transection (MCT) cases, most significantly related to complete SCIs by univariate analysis (P = 0.006), all showed complete SCIs when discharged, which makes it unsuitable for logistic regression. With MCT cases removed, univariate analysis was conducted again, then logistic regression. At last, only C5 spine injury (P = 0.024, OR = 0.241) was related to complete SCI. Cases with compression flexion injury mechanism (CFIM), most significantly related to motor complete SCIs by univariate analysis (P = 0.001), was also unsuitable for logistic regression for the same reason. At last, C3 spine injury (P = 0.033, OR = 0.068) and high energy injury (P = 0.033, OR = 14.763) were related to motor complete SCIs with CFIM cases removed. The results show that MCT and C5 spine injury are good predictors for complete/incomplete SCIs. CFIM, C3 spine injury and high energy injury are good predictors for motor complete/incomplete SCIs.

Spinal cord injury (SCI) is a devastating and debilitating condition that affects all regions of the world and is most common in the cervical region^1^. Outcome prediction of spinal cord injury is of great importance to both clinicians and researchers, while the outcomes in SCI exist on a continuum and are determined by a complex array of biomechanical and physiological factors^2^.

The regular emergency evaluations of traumatic cervical SCI include radiological evaluation and neurological assessment of motor and sensory function. The American Spinal Cord Injury Association (ASIA) Standards are commonly used to classify and evaluate neurological deficit after SCI in both clinical and research arenas. ASIA Standards is a reliable, valid and responsive instrument for descriptive and evaluative purposes in the adult SCI population in the acute care, rehabilitation and community settings. However, there are concerns with regard to the reliability of the early assessment of patients with acute traumatic SCI using the ASIA Standards. Spinal shock, medical instability, concomitant traumatic brain injury and coma are reportedly among the possible explanations for limitation of the ASIA Standards within 72 h after injury^3^,^4^,^5^.

Therefore, radiological evaluations within 72 h after injury have been explored as clinical predictors of neurological outcome in recent studies. CT and MRI examinations have been shown to have some prognostic values, but most of the past studies just conducted univariate analysis without considering confounding factors. The only two reviewed studies using univariate and multivariate analysis mainly just focused on the prognostic values of MRI evaluations^6^,^7^.

Thus, the purpose of this study was to investigate the prognostic values of clinical factors within 72 h after traumatic cervical SCI comprehensively.

## Results

Finally, 57 cases were included, 22 retrospective ones and 35 prospective ones. Detailed information is depicted in Table 1.

With “complete/incomplete injury” as the outcome measure, C5 spine injury (P = 0.018), C6 spine injury (P = 0.026), cord transection (P = 0.006, the lowest) and T2-MCC (P = 0.04) were significantly related to complete SCIs after univariate analysis (Table 2). All the cases with cord transection showed complete SCIs when discharged, which means that there will be a “zero” in the four numbers of the Crosstab by “cord transection” and “complete SCI (AIS = A)” (Table 3). Such situation is not suitable for logistic regression and cases with cord transection was removed for further analysis.

After all the cases with cord transection were removed, the rest cases were analyzed again. C5 (P = 0.021) and C6 (P = 0.042) spine injury were significantly related to complete SCI through unvariate analysis. After logistic regression, only C5 spine injury (P = 0.024, OR = 0.241) was negatively related to complete SCI (Table 4).

Because “MCC = 1” equals to “cord transection”, so we tried to conduct analysis of factors related to the outcome of complete SCI ignoring cord transection but not removing them. At last, C6 spine injury (P = 0.014, OR = 6.141) and T2-MCC (P = 0.021, OR = 47.206) were positively related to the outcome of complete SCI. (Table 5).

With “motor complete/incomplete injury” as the outcome measure, age over 50 (P = 0.027), high energy injury (P = 0.004), C3 spine injury (P = 0.01), distraction injury mechanism (P = 0.004), extension injury mechanism (P = 0.027), DE injury mechanism (P = 0.021), CF injury mechanism (P = 0.001, the lowest), cord swelling (P = 0.020) and cord hemorrhage or contusion (P = 0.038) were significantly related to motor complete SCIs after univariate analysis (Table 2). All the cases with CF injury mechanism showed motor complete SCIs when discharged, which also means that there will be a “zero” in the four numbers of the Crosstab by “cord transection” and “complete SCI (AIS = A)” (Table 6). Such situation is not suitable for logistic regression and cases with CF injury mechanism was removed for further analysis.

After all the cases with CF injury mechanism were removed, the rest cases were analyzed again. C3 spine injury (P = 0.037), high energy injury (P = 0.037) and cord swelling (P = 0.027) were significantly related to motor complete SCIs through univariate analysis. After logistic regression, C3 spine injury (P = 0.033, OR = 0.068) was negatively related to motor complete SCIs, and high energy injury (P = 0.033, OR = 14.763) was positively related to motor complete SCIs (Table 7).

## Discussion

All the cases with cord transection showed complete SCIs when discharged (6/57). And in theory, cord transection means complete separation of spinal cord into two segments and is sure to end up with complete spinal cord injury in spite of the highly unequal and sufficiently low group sizes. All the cases with CF injury mechanism showed motor complete SCIs when discharged (25/57). And also in theory, CF injury mechanism will cause the fracture of vertebral body, which is not the case for the other injury mechanisms. The anterior compression by the fractured vertebral body is likely to further contribute to the damage of spinal cord in contrast to the other injury mechanisms. Thus, we recommend that cord transection and CF injury mechanism should be the best predictors for complete SCI and motor complete SCI respectively.

### Prognostic values of subaxial cervical spine injury classifications

Allen Ferguson classification is, till now, the only classification system whose relationship with neurological outcome has been studied. And all the motor and sensory evaluations in the related studies were conducted using the guidelines established by the ASIA.

Kontautas *et al*.^8^ studied the relationship between neurological recovery and the patterns of cervical spine injury. They assessed neurological status of subjects after admission to hospital in the average of four hours after an accident. But there are concerns with regard to the reliability of the ASIA Standards for assessments earlier than 72 h after acute SCI as mentioned above. Nakashima *et al*.^9^ conducted a 12-month study of 146 patients with unstable subaxial cervical spine injuries and found that the stages of each injury patterns, but not the patterns based on Allen’s classification, are well-correlated with the neurological outcome. The 3-month study of 70 patients with cervical SCIs conducted by Shiozaki *et al*.^10^ also showed the similar results as the one by Nakashima *et al*.^9^. In addition, their study found that the patients with DE injury mechanisms had a higher rate of residual motor complete SCIs, even for the stage 1 and 2 cases.

Similar to the study conducted by Shiozaki *et al*.^10^, we found that DE injury mechanism was positively related to the outcome of motor complete SCI through univariate analysis. But only CF injury mechanism was found to be positively related to complete motor SCI at last. Taking into account of the high value of CF injury mechanism as a positive predictor for motor complete SCI, we consider the further classification of Group B1 (posterior disruption predominantly ligamentous) in AO-Magerl classification^11^ into B1.1 (posterior disruption predominantly ligamentous associated with transverse disruption of the disc) and B1.2 (posterior disruption predominantly ligamentous associated with type A fracture of the vertebral body) to be quite valuable for prognosis. Because the B1.1 injury can be classified as DF injury mechanism in Allen Ferguson classification, and the B1.2 injury as CF injury mechanism^12^. In the 2015 AOSpine cervical^13^, the subtype B2 (posterior tension band injury) and type C (B2) without rotation can be attributed to the Group B1 of AO-Magerl, but it is not further discussed whether a type A fracture of the vertebral body is accompanied or not. Maybe some further classifications should be considered.

In addition, we combined rotation and lateral compression into the conception of asymmetry spinal injury mechanism to investigate its relationship with outcome, but no relationship was detected. And our research failed to find any relationship of the injury morphology and disco-ligamentous complex integrity with outcome.

### Prognostic values of cervical spine injury segments

The segment of spine injury was classified by Furlan *et al*.^14^ into high-cervical (C3–C5) and low-cervical level (C6–C7) in their study. There were significant differences regarding the proportions of patients with high-cervical spine trauma among the patients with complete SCI (56%), incomplete SCI (82.69%) and spine trauma but without SCI (95.65%).

Similar to their study, our results showed that C6 spine injury was positively and C5 spine injury was negatively related to the outcome of complete SCI through univariate analysis when cord transection cases was excluded. But finally only C5 spine injury was negatively related after logistic regression. And C3 spine injury was negatively related to the outcome of motor complete SCI when cases with CF injury mechanism were excluded.

### Prognostic values of qualitative and quantitative MRI assessment

MRI can accurately depict both intrinsic changes in the spinal cord (i.e. cord hemorrhage and cord edema) and extrinsic compression of cord by herniated disc, epidural hematoma or bony fragments, which can help determine the cause and extent of the neurological deficits, the probable mechanism of injury, and the presence of spinal instability^15^. And some of its various qualitative and quantitative factors have been shown to be of prognostic values by recent studies.

Bondurant *et al*.^16^ recommended that the first MRI be performed 24–72 h post trauma without evidence-based ground, especially the “24 h”. And their own research defined all the signal patterns without mentioning “24 h”. Most of the following researches just adopted the time window of “within 72 h”. There was, till now, no evidence supporting a more precise guideline. And sagittal T2 images are the only sequences shown to have prognostic value.

Bozzo *et al*.^17^ recommended using four signal patterns (normal cord, single-level edema, diffuse edema, hemorrhage) in sagittal T2 images for prognostic evaluation. Normal cord: no signal abnormalities of the cord, despite neurological deficit (edema: hyperintensity within the cord, hemorrhage: hypointense within the cord with thin rim of hyperintensity).

The study of Ramon *et al*.^18^ also involved MRI signal patterns of compression, transection and contusion. Compression: severe obliteration of the spinal cord, with significant alteration of its morphology preventing detection of signal alterations such as bleeding. Transection: sagittal discontinuity of spinal cord. Contusion: normal images on T1WI, while on T2WI the spinal cord presents with a small central area of isointensity and a thick peripheral ring of high intensity. It was supposed to be included in the edema group by Bozzo *et al*.^17^. Cord swelling (focal widening of cord) was also adopted as a factor for prognosis by the study of Miyanji *et al*.^6^.

The study by Furlan *et al*.^19^ in 2007 evaluated the inter-rater and intra-rater reliability of 3 quantitative parameters from CT scan and MR images(CT-MCC, T1-MCC and T2-MSCC) of patients with cervical traumatic SCIs. The intra-rater correlation coefficients were considered as high with respect to agreement and consistency among raters. The inter-rater correlation coefficients were at a moderate level of agreement and consistency. Then the study by Furlan *et al*.^14^ in 2010 indicated that T1-MCC and T2-MSCC are responsive to changes in motor and sensory functions. However, CT-MCC provides inconsistent results that can result in misdiagnosis in the clinical setting.

And the study by Gupta *et al*.^7^ measured MCC on T2-weighted MRI, but not T1, which indicated that the presence of cord hemorrhage, T1-MCC and cord edema were best predictors of baseline neurological status at presentation, whereas baseline ASIA score and cord hemorrhage were best predictors of final neurological outcome. Considering the better prognostic value of T2-MCC. Our research measured T2-MCC and T2-MSCC.

Among all the literatures reviewed, only the study by Miyanji *et al*.^6^ and Gupta *et al*.^7^ analyzed factors through univariate and multivariable methods as we did.

In the research of Miyanji *et al*.^6^, three quantitative (T1WI-MCC, T2WI-MSCC, and lesion length) and six qualitative (intramedullary hemorrhage, edema, cord swelling, soft-tissue injury, canal stenosis, and disk herniation) imaging parameters were studied. The ASIA motor score was used as the outcome measure. They determined that the severity of T2-MSCC, intramedullary hemorrhage and cord swelling were key predictors of neurologic recovery after traumatic cervical SCI. We consider that ASIA motor score is highly related to the neurological level of SCI, which is inappropriate for outcome measure of this kind of study.

Gupta *et al*.^7^ analyzed three quantitative parameters (lesion length, T2-MCC and T2-MSCC) and nine qualitative parameters (vertebral fracture/subluxation, facet joint subluxation/dislocation, cord edema, hemorrhage/contusion, cord compression, epidural hemorrhage, traumatic disc herniation, ligamentous injury and pre/paravertebral collection) to determine the potential predictors of baseline ASIA score (at the time of MR study) and at the time of final follow up using multiple regression. The AIS was used as the outcome measure. They determined that the best predictors for baseline ASIA score were T2-MCC, cord edema and cord hemorrhage. For the final ASIA score, the best predictors were baseline AIS and cord hemorrhage. And ignoring the baseline AIS, we consider T2-MCC, cord edema and cord hemorrhage according to their research, especially the cord hemorrhage.

In our study, ignoring cord transection, T2-MCC was also found to be positively related to the outcome of complete SCI. Each case in our study showed cord edema, so we didn’t include it as a potential factor. However, the cord hemorrhage and contusion turned out not to be related to final outcome. Two possible explanations are considered. First, our cases were recruited from 2011 to 2015, the latest cases recruited in previous study were from 2009 to 2010^7^, the improvement in the emergency transportation and treatment of traumatic cervical SCI may be a reason for the result. Second, the MRI images of all the cases in our study were conducted in different hospitals in China. Considering the different filming parameters and time post injury, the signal change especially the hemorrhage may not be appropriate for the prediction of final outcome.

### Prognostic value of high/low energy injury

We also failed to find any relationship between canal stenosis before injury and final outcome. But high energy injury was positively related to motor complete SCIs after all the cases with CF injury mechanism were removed, which also means that low energy injury is negatively related. The pure canal stenosis may not be a predictor for outcome, it just increased the risk of cervical SCIs caused by low energy injuries, typically falling injury on the flat ground^20^. However, the cervical SCIs of cases with canal stenosis can be caused by both high and low energy injuries. Only low energy injury indicate higher chance for motor incomplete SCI, which was in accordance with the study of Boese *et al*.^21^, showing that the majority of patients presented with initial AIS grades of C (39.7%) and D (22.8%).

## Conclusions

**Figure 1:**
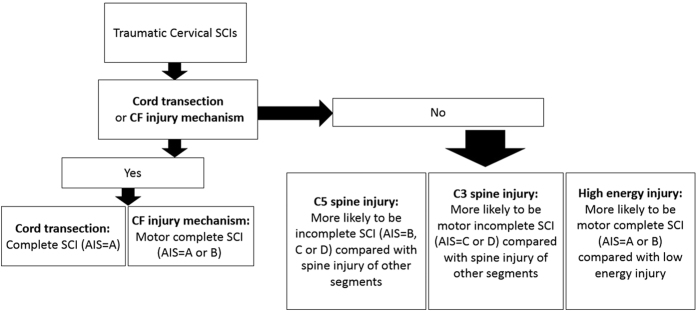
Algorithm for the outcome prediction of traumatic cervical SCIs.

The results of our research indicate that cord transection in MRI is a good predictor for complete SCI, and C5 spine injury is a good predictor for incomplete SCI. In addition, CF injury mechanism and high energy injury are good predictors for motor complete SCI. C3 spine injury is a good predictor for motor incomplete SCI. Finally, an algorithm is developed for the outcome prediction of traumatic cervical SCIs (Fig. 1).

## Methods

### Ethical approval

Ethical approval was given by the medical ethics committee of China Rehabilitation Research Center. Informed consent was obtained from the patients that were enrolled prospectively and most of the patients enrolled retrospectively who could be reached by phone. The informed consent of those who couldn’t be reached was waived for the retrospective nature by the ethics committee. Furthermore our study purely collected patients’ medical data for clinical analysis without filming videos or taking photos. And we promise that all the information collected will not be used for non-researching purposes and all the identifying information will be removed for publication.

### Study Population

Our study was initiated in April 2014. We retrospectively collected data through the medical records and radiological materials of traumatic cervical SCI inpatient patients of our department from as early as March 2011. Meanwhile, we prospectively collected the above data until December 2015.

Inclusion criteria: (1) Age ≥ 18. (2) Detailed CT and MRI materials 72 h within injury and before surgery should be accessible, and the outline and border of the spinal cord and spinal canal can be clearly recognized in the mid and parasagittal T2-weighted MRI images. (3) The patients should be transferred by ambulance after injury. (4) The patients should receive surgical treatments, and the images after surgery should show good reduction of spinal column and sufficient decompression of spinal cord. (5) The patients should receive rehabilitation treatment for more than 6 months after injury.

Exclusion criteria: (1) Congenital disease of spine and spinal cord. (2) Infectious and neoplastic disease of spine and spinal cord. (3) Accompanied injuries of other spine and spinal cord segment or craniocerebral injuries. (4) Unable to cooperate with neurological assessment for various reasons including psychiatric disorders; (5) Serious medical illnesses, such as heart or kidney failure.

### Outcome Measures

The following ASIA Impairment Scale (AIS) designation is used in grading the degree of impairment^22^:

A = Complete. No sensory or motor function is preserved in the sacral segments S4–S5.

B = Sensory incomplete. Sensory but not motor function is preserved below the neurological level and includes the sacral segments S4–S5, AND no motor function is preserved more than three levels below the motor level on either side of the body.

C = Motor incomplete. Motor function is preserved below the neurological level, and more than half of key muscle functions below the single neurological level of injury have a muscle grade less than 3 (Grades 0–2).

D = Motor incomplete. Motor function is preserved below the neurological level, and at least half (half or more) of key muscle functions below the NLI have a muscle grade >3.

E = Normal. If sensation and motor function as tested with the ISNCSCI are graded as normal in all segments, and the patient had prior deficits, then the AIS grade is E. Someone without a SCI does not receive an AIS grade.

The motor and sensory evaluations were conducted using the guidelines established by the ASIA. And final outcome of AIS when discharged was divided into dichotomous variables by two methods for the sake of logistic regression, namely “complete (AIS = A)/incomplete SCI (AIS ≠ A)” and “motor complete (AIS = A or B)/incomplete SCI (AIS ≠ A and B)”.

### Data extraction

The following information was collected from medical records: age when injured, gender, medical history of ankylosing spondylitis (AS), spinal stenosis basis before injury, injury energy (high energy: traffic accident injury, high falling injury, crushing injury by heavy objects, low energy injury: falling injury from flat ground), time from injury to surgery, surgical approach (AIS at admission is not included because different patients was admitted at various time post injury).

**Figure 2:**
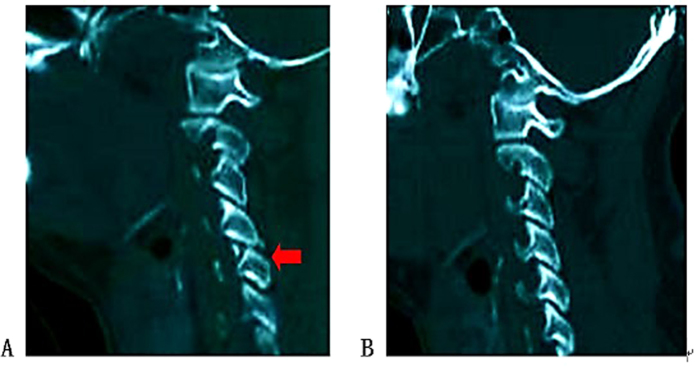
Asymmetry facet joints. (**A**) fractured right superior articular process of C6; (**B)** normal left superior articular process of C6.

**Figure 3:**
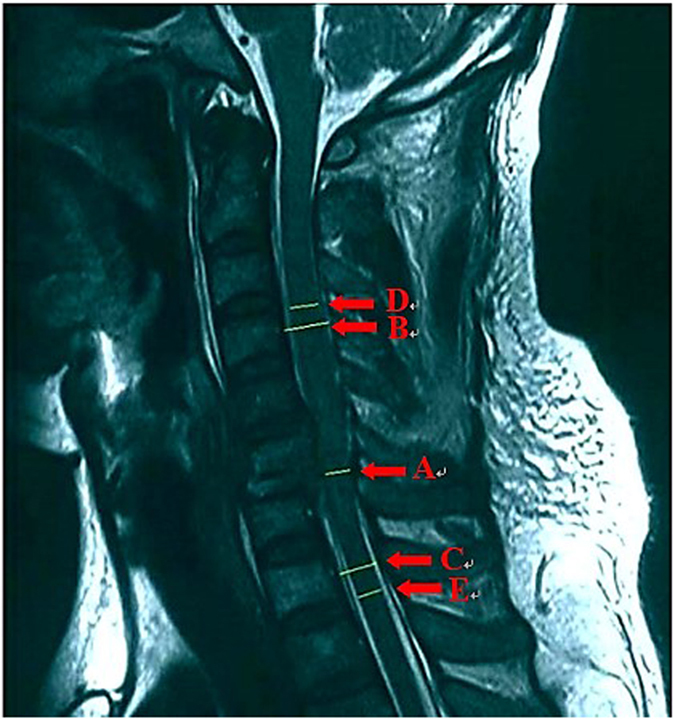
Quantitative assessment of mid-sagittal T2 MRI. T2WI-MCC = 1-A*2/(B + C), T2WI-MSCC = 1-A*2/(D + E).

**Figure 4:**
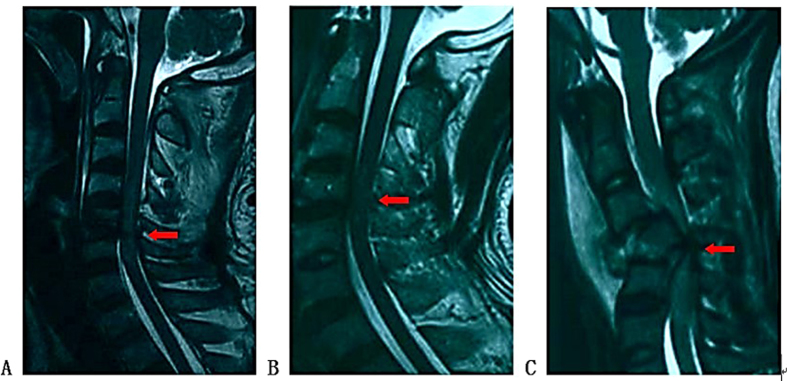
Qualitative assessment of sagittal T2 MRI. (**A**) Cord hemorrhage with cord swelling just below the compressed area; (**B**) Cord contusion; (**C)** Cord transection.

The following information was collected from CT and MRI images: injury segment of cervical spine (Cx/x+1 dislocation and Cx compression or burst fracture were all recorded as Cx spine injury), asymmetry spinal injury mechanism (asymmetry compression of the spinal column or asymmetry morphology of facet joints) (Fig. 2A and B), Allen Ferguson mechanistic classification (injury mechanism: compression flexion(CF), distraction flexion(DF), compression extension(CE), distraction extension(DE), axial compression(AC), lateral compression(LC)), SLIC (injury morphology and disco-ligamentous complex integrity), 2015AO subaxial cervical spine traumatic injury classification system (termed “2015AO cervical” for short in the following text) morphology type, T2-MSCC (T2-weighted MRI-maximal spinal cord compression), T2-MCC(T2-weighted MRI-maximal canal compromise)(Fig. 3), MRI signal of cord hemorrhage (hypointense on T2WI with thin rim of hyperintensity, 72 h within injury)(Fig. 4A), contusion (isointense on T2WI with thin rim of hyperintensity, 72 h within injury)(Fig. 4B), swelling(focal widening of cord)(Fig. 4A) and transection(sagittal discontinuity of spinal cord)(Fig. 4C).

The AO-Magerl classification defined type C injury as anterior and posterior element injuries with rotation^11^. The 2015 AOSpine cervical changed the definition of type C into translational injury in any axis^13^. The conception of rotation is no longer mentioned. And the SLIC morphology classification has a translational/rotational type^23^, Allen Ferguson include a LC injury mechanism^12^. Therefore, we combined rotation and lateral compression into the conception of asymmetry spinal injury mechanism to investigate its relationship with outcome.

**Figure 5:**
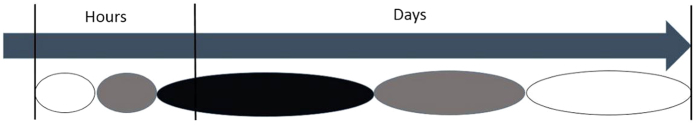
Possible evolution of hematoma in T2-weighted image.

Hematoma signal in T2-weighted images will change from bright (hyperintensity) to dark (hypointensity) during hours, and change back from dark (hypointensity) to bright (hyperintensity) during days^24^, which might result in an interface of isointensity signal during both hours and days (Fig. 5). Thus both contusion and hemorrhage might just be the MRI signal change of hematoma at different time, but not two different kinds of injury type. So we also tried to combine the two signals into one to investigate its prognostic value except for analyzing them separately (i.e. 3 parameters: “hematoma”, “contusion” and “hematoma or contusion”).

**Figure 6:**
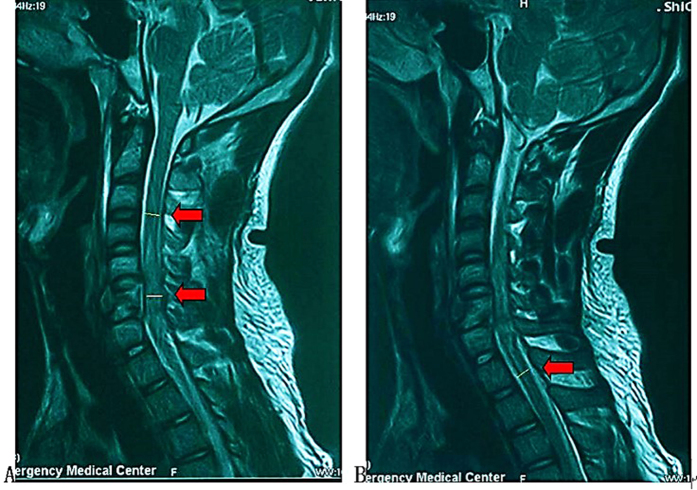
Supplementary measurement for T2-MSCC. (**A**) Measurement of MSCC can’t be finished in the mid-sagittal T2 image. (**B**) Parasagittal image is selected to supplement the measurement.

When the cervical spine is slightly laterally flexed or the spinal cord is compressed to one lateral side of the spinal canal. The measurement of MCC or MSCC can’t be finished just in the mid-sagittal T2-weighted MRI image, which have not been reported in previous literatures. We adopted the adjacent para-sagittal image to supplement the measurement (Fig. 6).

### Statistical analyses

Comparisons were made between two groups. Patients with complete SCI (ASIA grade A) are compared with patients with incomplete SCIs (ASIA grade B, C, or D), and patients with motor complete injury (ASIA grade A or B) with motor incomplete injury (ASIA grade C or D). Data were analyzed using univariate and multivariable methods.

The qualitative parameters (gender, medical history of AS, spinal stenosis basis before injury, high injury energy, asymmetry spinal injury mechanism, disco-ligamentous complex integrity, hematoma, contusion, hematoma or contusion and swelling signals in MRI) was analyzed as dichotomous variables (ie, findings either present or absent).

The categorical parameters (surgical approach, injury segment of cervical spine, neurological level of injury, Allen Ferguson mechanistic classification, 2015AO cervical morphology type) were first analyzed first as a whole. If found not related to the outcome in the univariate analysis, they will be changed into dichotomous variables by the presence of a single category or not (such as: anterior approach or not, C5 spine injury segment or not, CF injury mechanism or not, 2015AO cervical morphology C(B2) type or not) and analyzed again.

The quantitative parameters (age when injured, time from injury to surgery, T2-MSCC(T2-weighted MRI-maximal spinal cord compression), T2-MCC(T2-weighted MRI-maximal canal compromise) were first analyzed as valued metrics. If found not related to the outcome in the univariate analysis, they will be changed into dichotomous variables (such as age when injured was ≥50 or not, time from injury to surgery was ≤24 h or not) and analyzed again.

The Mantel-Haenszel x^2^ test or the Fisher exact test were used to evaluate whether there was a difference between the two groups in terms of the frequency of all the dichotomous or categorical variables. Independent T test was use to compare the valued metrics between the two groups. Variables significantly related to the compared two groups were analyzed through logistic regression to find out the best model for predicting neurological status when discharged. All the significance was set at the 5% level.

## Additional Information

**How to cite this article:** Qiu, Z. *et al*. Clinical Predictors of Neurological Outcome within 72 h after Traumatic Cervical Spinal Cord Injury. *Sci. Rep.* **6**, 38909; doi: 10.1038/srep38909 (2016).

**Publisher's note:** Springer Nature remains neutral with regard to jurisdictional claims in published maps and institutional affiliations.

## Figures and Tables

**Table 1:** Detailed information of all the cases.

Factors	Number	Factors	Number
*Gender* Male/Female	47/10	*Asymmetry spinal injury mechanism*	
		With/Without	6/51
*Age when injured* <50/≥50	42/15	*Cord contusion* With/Without	14/43
*Cause of injury*		*Cord hemorrhage* With/Without	22/35
*High energy /Low energy*	52/5	*Cord swelling* With/Without	34/23
*AS* With/Without	4/53	*Cord transection* With/Without	6/51
*Spinal canal stenosis basis*		*Neurological level*	
With/Without	6/51	C3/C4/C5/C6/C7	5/36/9/6/1
*Time from injury to surgery*		*Injury mechanism*	
≤24 h/>24 h	13/43	CF/DF/AC/CE/DE/LC	25/16/3/4/7/2
*Surgical approach selected*		*2015AO cervical*	
Anterior/Posterior/Combined	41/9/7	A4/B2/B3/C(B2)/C(B3)	5/1/3/40/8
*Cervical spine injury segment*		*AIS when discharged* A/B/C/D	26/20/9/2
C3/C4/C5/C6	6/12/25/14		
*SLIC:*		*Sagittal T2WI*	*Mean* ± *SD*
Morphology + DLC integrity		MCC	0.51 ± 0.20
4 + 2/3 + 2/2 + 0	49/3/5	MSCC	0.27 ± 0.31

**Table 2:** Univariate analysis for all the cases.

	Univariate analysis	Variables not appropriate for logistic regression
AIS = A	C5 spine injury (P = 0.018)	Cord transection (P = 0.006) (+)
or not	C6 spine injury (P = 0.026)	
	*Cord transection (P = 0.006)*	
	T2-MCC (P = 0.04)	
AIS = A or B	Age over 50 (P = 0.027)	CF injury mechanism (P = 0.001) (+)
	High energy injury (P = 0.004)	
or not	C3 spine injury (P = 0.01)	
	Distraction injury mechanism (P = 0.004)	
	Extension injury mechanism (P = 0.027)	
	DE injury mechanism (P = 0.021)	
	*CF injury mechanism (P* = *0.001)*	
	Cord swelling (P = 0.020)	
	Cord hemorrhage or contusion (P = 0.038)	

**Table 3:** Crosstab of cord transection and outcome of complete SCI for all the cases.

		Cord transection		Summation
		Without	With	
AIS = A	No	31	0	31
when discharged	Yes	20	6	26
Summation		51	6	57

**Table 4:** Variables significantly related to complete SCI with cord transection cases removed.

	Variables significantly related
Univariate analysis	C5 spine injury (P = 0.021)
	C6 spine injury (P = 0.042)
Logistic regression	C5 spine injury (P = 0.024, OR = 0.241) (−)

**Table 5:** Variables significantly related to complete SCI ignoring cord transection for all the cases.

	Variables significantly related
Univariate analysis	C5 spine injury (P = 0.018)
	C6 spine injury (P = 0.026)
	T2-MCC (P = 0.04)
Logistic regression	C6 spine injury (P = 0.014, OR = 6.141) (+)
	T2-MCC (P = 0.021, OR = 47.206) (+)

**Table 6:** Crosstab of CF injury mechanism and outcome of motor complete SCI for all the cases.

		CF injury mechanism		Summation
		Without	With	
AIS = A or B	No	11	0	11
when discharged	Yes	21	25	46
Summation		32	25	57

**Table 7:** Variables significantly related to motor complete SCI with CF injury mechanism cases removed.

	Variables significantly related
Univariate analysis	High energy injury (P = 0.037)
	C3 spine injury (P = 0.037)
	Cord swelling (P = 0.027)
Logistic regression	C3 spine injury (P = 0.033, OR = 0.068) (−)
	High energy injury (P = 0.033, OR = 14.763) (+)
